# Application of a Terrestrial LIDAR System for Elevation Mapping in Terra Nova Bay, Antarctica

**DOI:** 10.3390/s150923514

**Published:** 2015-09-16

**Authors:** Hyoungsig Cho, Seunghwan Hong, Sangmin Kim, Hyokeun Park, Ilsuk Park, Hong-Gyoo Sohn

**Affiliations:** School of Civil and Environmental Engineering, Yonsei University, Seoul 120-749, Korea; E-Mails: f15kdaum@yonsei.ac.kr (H.C.); hotaeim@yonsei.ac.kr (S.H.); netgo82@yonsei.ac.kr (S.K.); bear0514@gmail.com (H.P.); moncher@yonsei.ac.kr (I.P.)

**Keywords:** terrestrial LIDAR, Antarctica, GNSS, point cloud, co-registration, georeferencing, DEM, topographic map

## Abstract

A terrestrial Light Detection and Ranging (LIDAR) system has high productivity and accuracy for topographic mapping, but the harsh conditions of Antarctica make LIDAR operation difficult. Low temperatures cause malfunctioning of the LIDAR system, and unpredictable strong winds can deteriorate data quality by irregularly shaking co-registration targets. For stable and efficient LIDAR operation in Antarctica, this study proposes and demonstrates the following practical solutions: (1) a lagging cover with a heating pack to maintain the temperature of the terrestrial LIDAR system; (2) co-registration using square planar targets and two-step point-merging methods based on extracted feature points and the Iterative Closest Point (ICP) algorithm; and (3) a georeferencing module consisting of an artificial target and a Global Navigation Satellite System (GNSS) receiver. The solutions were used to produce a topographic map for construction of the Jang Bogo Research Station in Terra Nova Bay, Antarctica. Co-registration and georeferencing precision reached 5 and 45 mm, respectively, and the accuracy of the Digital Elevation Model (DEM) generated from the LIDAR scanning data was ±27.7 cm.

## 1. Introduction

Antarctica is a highly valuable for representing global climate change trends; thus, extensive research has investigated and analyzed its environment [1,2]. In particular, elevation mapping to monitor changes in glaciers and ice sheets is important in tracking climate changes. Many glaciologists have used Digital Elevation Models (DEMs) to investigate the topography of Antarctica [3,4,5,6].

The remote location and harsh weather conditions of Antarctica make direct ground elevation observations difficult. For this reason, satellite-based technologies that can obtain periodic surface information on inaccessible areas have been widely used to produce DEMs of Antarctica [7]. Specifically, satellite altimetry, optical satellites, and Interferometric Synthetic Aperture Radar (InSAR) have all been utilized for this purpose. Satellite altimetry can directly measure surface elevations over a large area using radar and laser technologies [8,9,10,11,12]. Moreover, optical and Synthetic Aperture Radar (SAR) satellites also can acquire elevation information for a large glaciated region based on photogrammetry and the InSAR technique, respectively [7,13,14,15,16,17]. 

Satellite-based technologies can be applied in the large-scale research of glaciers and ice sheets because of their high productivity, but DEMs from these approaches are limited in terms of accuracy and spatial resolution. On the other hand, the Global Navigation Satellite System (GNSS) approach provides the direct 3D coordinates of the GNSS receiver with a high degree of accuracy. This method has been widely used in small-scale studies on research areas such as Antarctic plate motion and deformation and accuracy evaluation of satellite observations [18,19,20,21]. Despite the high accuracy of GNSS observations, however, achieving high-density elevation information across a large area is labor intensive.

With recent increasing demand for high-resolution DEMs, the airborne and terrestrial Light Detection and Ranging (LIDAR) systems have received attention from various fields, such as forestry [22,23], urban mapping [24,25,26], and disaster management [27,28,29]. Airborne LIDAR, which linearly profiles target areas using a laser scanning system, can obtain a DEM with decimeter-level resolution [30,31,32,33]. However, the low accessibility of Antarctica makes operation of the airborne LIDAR impractical. Moreover, surface characteristics and weather conditions can lead to unpredictable errors, such as those due to poor signal return and rolling of the LIDAR platform, and important surface information may be missed [34,35].

Terrestrial LIDAR, which observes the 3D shape of land surfaces with centimeter-level accuracy, is widely used for various applications [36,37,38,39,40]. Although terrestrial LIDAR functions similarly to a total station, it rapidly emits laser pulses and produces detailed 3D information on objects [41]. In addition, color images collected by a digital camera mounted on the terrestrial LIDAR system can be merged with the laser scanning data. However, despite the high productivity and accuracy of terrestrial LIDAR observations, the actual use of terrestrial LIDAR in the harsh weather conditions of Antarctica is difficult. Accordingly, sufficient planning for LIDAR observation is required [42]. However, no formulaic procedure has been suggested for the operation of the terrestrial LIDAR system in extreme weather conditions [42]. This study therefore proposes and demonstrates practical solutions for terrestrial LIDAR observation, especially for the operation, co-registration, and georeferencing procedures, to obtain high-resolution elevation data in the harsh environment of Antarctica.

## 2. Experimental Section

### 2.1. Study Area

**Figure 1:**
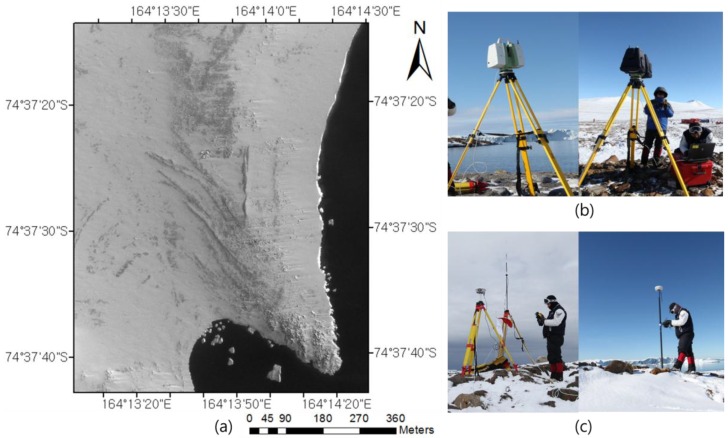
Research site and field survey: (**a**) location map; (**b**) terrestrial LIDAR observation; (**c**) Real-Time Kinematic (RTK) GNSS observation.

In 2006, the Korean government created a long-term plan to build a second research station in Antarctica to enhance scientific capabilities and promote collaboration among Antarctic communities. Korea’s first Antarctic station, the King Sejong Station (62°13′ S, 58°47′ W), located in Barton Peninsula, King George Island, is used primarily to investigate the behavior of energy particles entering Earth’s magnetic field from space. The site of the second station at Terra Nova Bay in northern Victoria Land was chosen from ten candidate locations of scientific interest. The research team conducted an intensive field survey of the area in order to build the station. Terrestrial LIDAR and GNSS observations were conducted over 13 days around the new station, Jang Bogo Station, in February 2011. The geographic coordinates of the research site were 74°37′24″ S, 164°13′42″ E, and the area was approximately 459,200 m^2^. Figure 1 shows the location map of the research site.

**Figure 2:**
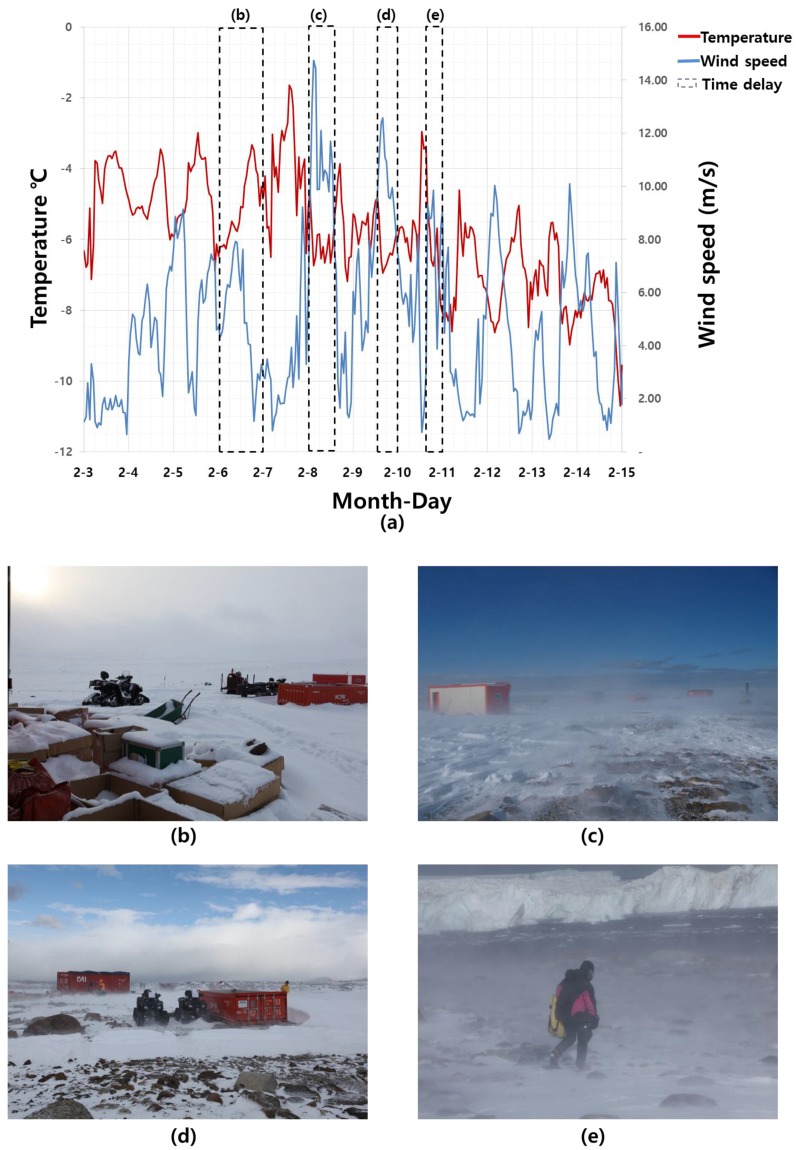
Weather conditions in Antarctica during the research period: (**a**) temperature and wind speed; (**b**) heavy snow on 02/06/2011; (**c**–**e**) blizzards on 02/08/2011, 02/10/2011, and 02/11/2011, respectively.

Figure 2 depicts the weather conditions in Antarctica during the field survey. As shown in the figure, the temperature fluctuated between −8.6 and −1.5 °C, and the wind speed varied, reaching up to 14.74 m/s. Harsh weather conditions, such as heavy snow and blizzards, caused time delays for field surveying. On February 6, 2011 (Figure 2b), despite relatively warm temperatures and low wind speeds, surveyors could not move because thick snow covered the surface of the research site. Additionally, because of blizzards with strong winds and heavy snow on the mornings of February 8 (Figure 2c), and the afternoons of February 10 (Figure 2d), and 11 (Figure 2e), it was impossible to perform GNSS and LIDAR surveys.

### 2.2. Overview of Methodology

Operation, co-registration, and georeferencing procedures directly affect the final accuracy of terrestrial LIDAR observations [43]. Because of the cold and irregularly windy weather conditions of Antarctica, LIDAR scanning at each station should be conducted within a short period. At the same time, both the stability during scanning and the laser reflectivity for snow-covered surfaces should be guaranteed. For this reason, terrestrial LIDAR specifications, such as effective range, scan rate, intensity of the emitted laser, and operating temperature, are the major concerns for effective and time-efficient scanning. Additionally, because the low temperatures of Antarctica can impede LIDAR operation, preliminary tests to verify the scanning stability of a LIDAR system in harsh weather conditions are essential. Based on the test results, practical solutions to maintain the necessary temperature to keep the LIDAR system continuously in the operational phase must be pursued.

Co-registration is the process of transforming multiple LIDAR scanning data into a common reference coordinate system. For high precision of co-registration, a sufficient number of matched points in pairwise point clouds is required in addition to the geometry. Co-registration method and stability of scanners and targets at the scan site also affect the final accuracy of multi-scanned data. Moreover, the occlusion area of a scanning site must be minimized while the scanning coverage is maximized. In this regard, square planar targets were mainly utilized, and subsidiary target-free techniques were applied to improve time efficiency and co-registration precision.

In LIDAR point cloud data processing, georeferencing follows co-registration. A procedure that converts the relative coordinates of a point cloud into an absolute coordinate system, georeferencing is one of the most important processes in accurate topographic mapping with terrestrial LIDAR data. For a robust georeferencing process, a significant number of fixed control points whose 3D coordinates are known is required. However, since the terrain is very complex and irregular in Antarctica, it is difficult to extract well-defined 3D coordinates of feature points from LIDAR scanning data. To acquire accurate 3D coordinates of feature points, we developed a practical georeferencing module consisting of a GNSS receiver and planar target. The modules were installed during LIDAR scanning and used for georeferencing after the scanning. Triangulated Irregular Network-based (TIN-based) linear interpolation was applied to generate a DEM from the processed point cloud [44,45].

To evaluate the performance of our proposed solutions for LIDAR scanning, checkpoints were acquired using the Real-Time Kinematic (RTK) GNSS technique, which can obtain accurate 3D coordinates of a GNSS receiver within a very short period of time. To obtain the 3D coordinates of several thousand checkpoints, three GNSS receivers for RTK GNSS data acquisition (one for the base station and two for the rover receiver) were utilized. The detailed methodology of each procedure is explained in the following sections.

### 2.3. Operational Phase

The first critical issue for operation is to determine whether the terrestrial LIDAR system can function in the harsh environment of the research site. As operation in subzero temperatures is not guaranteed [46], a feasibility test should be conducted to determine functionality in such conditions. Another key consideration in achieving efficiency and high quality of observed point cloud data is the type of terrestrial LIDAR. Terrestrial LIDAR systems generally use one of two types of measurement schemes: A Direct Time of Flight (TOF) or an Amplitude-Modulated Continuous Wave (AMCW).

Each method has its own effective range, scan rate, and accuracy [47,48]. Direct TOF terrestrial LIDAR systems can cover a range of up to several hundred meters and are suitable for outdoor observation. However, in order to generate and emit pulses with an intended intensity, direct TOF systems require more time to obtain high-density point cloud data than do AMCW systems [48]. On the other hand, the effective range of AMCW systems is generally limited to 100 m because the modulated signal is too weak to measure the phase shift between the emitted and received signals at greater distances. AMCW systems do have the advantage of a higher scan speed and thus are used for indoor observation [48]. Because of such differences in terrestrial LIDAR systems, careful selection of a suitable system type is important for observation productivity and accuracy.

Considering the outdoor, harsh conditions of Antarctica, a direct TOF system, Leica’s ScanStation C10, was selected for its high intensity and effective range of 300 m at 90% albedo and 100 m at 18% albedo. Moreover, the reflectance of the laser beam at a wavelength of 532 nm was suitable for observing snow surfaces [37]. Detailed specifications of the ScanStation C10 are summarized in Table 1.

**Table 1:** ScanStation C10 Specifications [46].

ScanStation C10		Specification
Type		Pulsed
Wavelength		532 nm
Range		300 m at 90% albedo, 134 m at 18% albedo (minimum range 0.1 m)
Scan rate		Up to 50,000 points/s (maximum instantaneous rate)
Accuracy of single measurement *	Position	6 mm
	Distance	4 mm
	Angle	60 µrad
Field of view	Horizontal	360° (maximum)
	Vertical	270° (maximum)
Weight		13 kg
Size (depth, width, height)		238, 358, 395 mm
Operating temperature		0 °C to +40 °C
Storage temperature		−25 °C to +65 °C
Camera		Auto-adjusting, integrated high-resolution digital camera with zoom video

* At 1–50 m range, one sigma.

In addition, since unpredictable weather conditions such as strong winds and heavy snow can lead to errors, the system must have an adequate scan rate and operating temperature range. The scan rate of the ScanStation C10, equipped with a Smart X-Mirror™ and a vertically rotating mirror on a horizontally rotating base, was quite sufficient for observations in Antarctica [46]. Moreover, the LIDAR has a dual-axis compensator to correct the horizontal angle when the device is inclined [49]. In addition, the high-resolution camera mounted on the terrestrial LIDAR provided RGB information combined with 3D point data. However, the operating temperature range was between 0 and 40 °C. Because this range is the most critical factor for stable operation in the field, additional usability verification of the system was essential.

**Figure 3:**
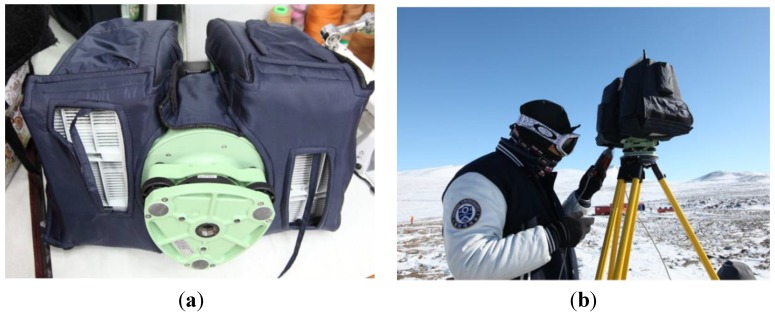
(**a**) Devised lagging cover for the terrestrial LIDAR; (**b**) *in situ* usage of the cover in Antarctica.

To verify the functionality of the system at low temperatures, feasibility tests were conducted in an artificial chamber in which the temperature could be dropped to −15 °C. The results of the experiments indicated that the terrestrial LIDAR system failed to operate below −10 °C. Although the battery of the device drained quickly between −10 and 0 °C, the LIDAR system operated well in this range after a preheating time of about seven minutes. However, below −10 °C, the system was unable to emit its laser even after sufficient preheating. To resolve this problem in the field, a lagging cover was designed, containing six heating packs to raise the temperature of the instrument. Since the terrestrial LIDAR system consumes substantial energy and requires preheating for operation in low temperatures, a portable generator was additionally required. The devised lagging cover is shown in Figure 3a and its usage in the field in Figure 3b.

### 2.4. Co-Registration and Georeferencing Phases

Co-registration can be classified into two categories: target-based and target-free approaches. The target-based co-registration approach utilizes user-made artificial targets and is the most popular method because of its convenience and accuracy [43]. Paper, paddle, and sphere targets have been widely used for co-registration in terrestrial LIDAR systems. Co-registration using sphere targets has shown the best performance in both indoor and outdoor environments [50]. When the use of artificial targets is impractical, co-registration is usually performed using natural feature points extracted from overlapped point clouds [51]. Since erroneous extraction of the feature points can cause severe reduction of co-registration accuracy, feature points should be carefully selected by an expert. When there is no target or feature point for co-registration, the Iterative Closest Point (ICP) algorithm, which conducts co-registration by minimizing the locational inconsistency between a pair of point sets, is commonly used [52,53].

Considering the pros and cons of the co-registration methods and the environment of Antarctica, target-based co-registration using the square planar targets (7.62 × 7.62 cm) as matching points was selected and conducted. The research team also avoided strong winds over 10 m/s and secured tripods with ropes and turnbuckles to achieve sufficient precision of co-registration. When unpredictable strong winds affected the stability of artificial targets, we extracted matching points from natural feature points in overlapped point clouds to apply the target-based algorithm. The ICP algorithm was additionally applied to improve co-registration precision.

**Figure 4:**
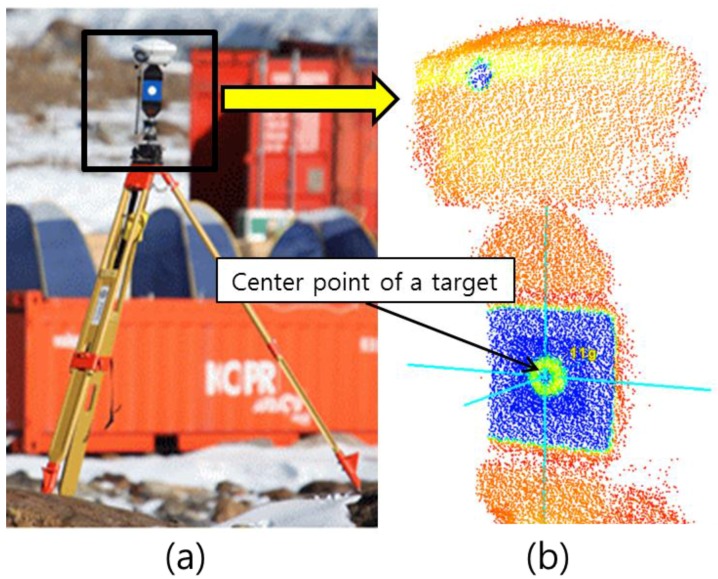
Devised target module for georeferencing of scanning data: (**a**) *in situ* utilization; (**b**) configuration after scanning (3D coordinates of the center point of the target can be identified accurately in a point cloud).

Even after co-registration is complete, a merged point cloud still has a relative coordinate system. Georeferencing is required to convert this system into an absolute coordinate system. During georeferencing, control points whose absolute 3D positions are known are needed. To overcome the problems presented by strong winds and obtain accurate 3D coordinates of control points for georeferencing, a special georeferencing module that combined a square planar target with a GNSS receiver was devised (Figure 4). The devised module can be used to transform a relative point cloud coordinate system into the World Geodetic System 1984 (WGS84). As shown in Figure 4b, the center coordinate of a square planar target of 7.62 × 7.62 cm can be identified accurately from the point cloud data. This square planar target can be replaced by another artifact, such as a circular planar target developed by UNAVCO [54]. The center points of the planar targets were used as the control points for georeferencing, and the 3D coordinates of the control points were calculated based on the observed coordinates of the GNSS receivers. Sufficient numbers of the modules were installed during LIDAR scanning to achieve geometry for precise georeferencing.

### 2.5. Digital Elevation Model (DEM) Generation

**Figure 5:**
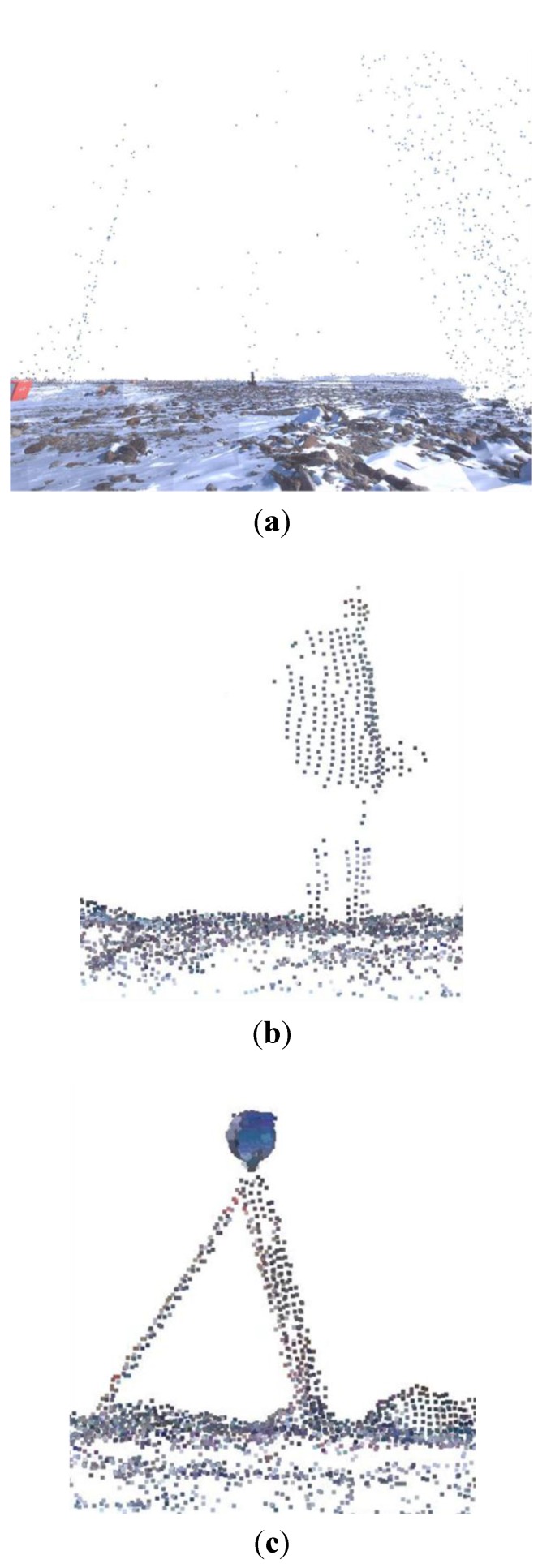
Filtered noise points for TIN modeling: (**a**) snow; (**b**) workers; (**c**) devices.

To produce topographic maps of the research site, we consecutively generated a high-resolution Triangulated Irregular Network (TIN), grid-based DEM, and contour lines from the georeferenced point cloud data. Prior to all data processing, noisy point data, such as those obtained from snow, fieldworkers, and field devices, were filtered out to improve the quality of the final products. Figure 5 shows examples of noisy data points.

**Figure 6:**
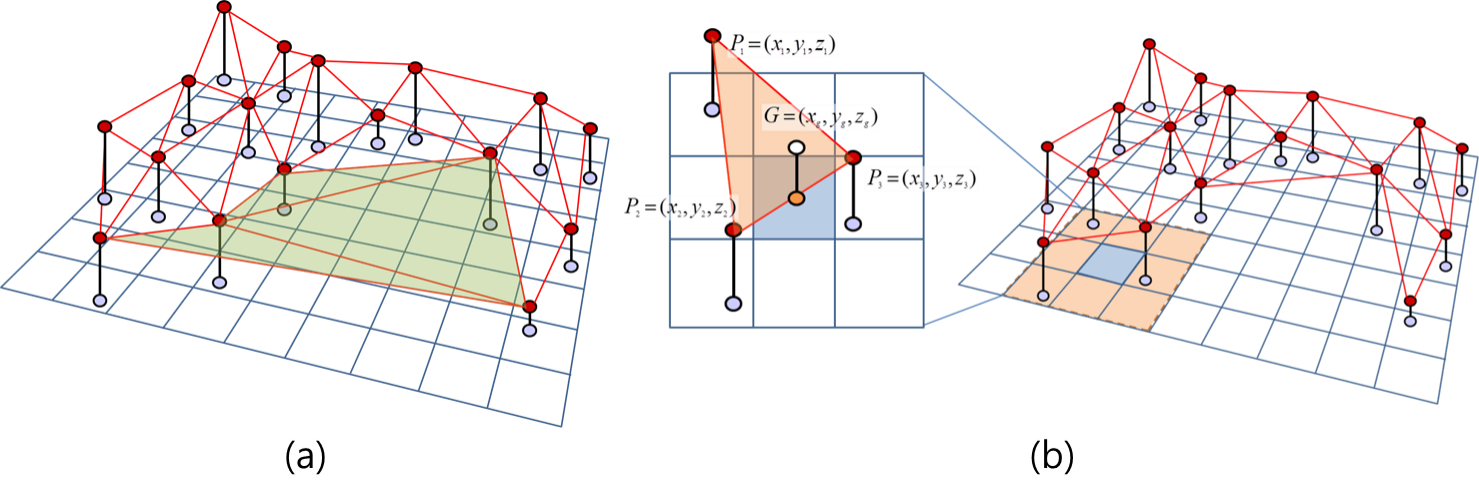
Description of data processing for DEM generation: (**a**) TIN model (red circles are points of an observed point cloud, red lines are generated TIN, and green triangles represent the removed TIN elements with long edges and consisting of a large area around the point cloud); (**b**) TIN-based interpolation for DEM generation: blue lines are DEM grids, and the 3D coordinates of a white circle (*G*) are *xy* location (*x_g_*, *y_g_*) and interpolated height (*z_g_*) from the vertices of the TIN element (*P*_1_, *P*_2_ and *P*_3_).

After filtering out noise, a TIN model can be created from the filtered point data. However, as shown in Figure 6a, unnecessary triangles that cause distortion in the final topographic map can be generated along the edge of point cloud. To improve the final quality of the DEM generated from the TIN, triangles with long edges and containing large areas are removed. A high-resolution DEM and contour lines can then be generated using the TIN-based linear interpolation technique [44] (Figure 6b). As shown in the figure, the height (*z_g_*) of each grid cell (*G*) can be calculated from the vertices of each TIN element (*P*_1_, *P*_2_ and *P*_3_). Equation (1) is used for the TIN-based linear interpolation, as follows:

(1) $$z_{g}=\frac{\sum_{i=1}^{3}\left(\frac{z_{i}}{d_{i}}\right)}{\sum_{i=1}^{3}\left(\frac{1}{d_{i}}\right)}$$

where *z_j_* is the height of the vertices of each TIN element, and *d_j_* is the horizontal distance between each grid cell (*x_g_*, *y_g_*) and vertex (*x_i_*, *y_i_*), calculated by Equation (2) as follows:

(2) $$d_{i}=\sqrt{{\left(x_{g}-x_{i}\right)}^{2}+{\left(y_{g}-y_{i}\right)}^{2}}$$

### 2.6. Accuracy Assessment

**Figure 7:**
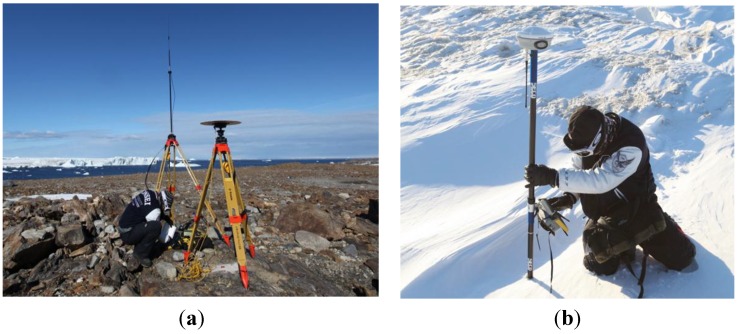
GNSS observation at the research site: (**a**) installation of a base station and radio modem for RTK GNSS; (**b**) *in situ* observation using a rover.

To evaluate the accuracy of the final terrestrial LIDAR data, checkpoints obtained from RTK GNSS observations were utilized. It is well known that the RTK GNSS method can acquire 3D coordinates of a point with high accuracy and precision [55,56]. To perform RTK GNSS, at least two GNSS receivers are required as a rover receiver and base station. A rover GNSS receiver rapidly measures the 3D coordinates of observed points. A receiver at the base station transmits real-time correction information to the rover using a radio modem. In this research, two rovers, one base station, and a TDL-450 UHF radio modem were operated to calculate 3D coordinates of checkpoints with approximately 20 m line spacing. Figure 7 depicts the RTK GNSS observation during field research. Since RTK GNSS is a real-time technique and the rover receivers can observe 3D coordinates of points within a few seconds, the influence of wind may be negligible. However, base station movement might affect observation accuracy. Our research team therefore continuously monitored the movement of the base station to minimize this effect.

Accuracy assessment of the processed point cloud was conducted based on the nearest neighborhood scheme. For each checkpoint, the nearest point was found from the LIDAR data, and the Root Mean Square Error (RMSE) between the two points was calculated. The RMSE was calculated by Equation (3), as follows:

(3) $$RMSE=\sqrt{\frac{1}{N}\sum_{i=1}^{N}{\left(z_{i}^{cp}-z_{i}^{pc}\right)}^{2}}$$

where *N* is the number of the matched points, $z_{i}^{cp}$ is the height of the *i*th checkpoint, and $z_{i}^{pc}$ is the height of the *i*th closest point to the checkpoint inside the point cloud.

## 3. Results and Discussion

### 3.1. Data Acquisition

Table 2 lists all activities performed during the research period from 3–14 February 2011 based on a pre-activity plan, as the core of fieldwork in the harsh Antarctic environment, which included a backup plan. GNSS observations and terrestrial LIDAR scanning were conducted for five days and two days, respectively. Fieldwork could not be performed at all on 6 February (Figure 2b) because of the heavy snow. Blizzards on the morning of February 8 (Figure 2c) and the afternoons of 10 February (Figure 2d) and 11 February (Figure 2e) delayed GNSS observation and LIDAR scanning. Even with irregular strong winds in the afternoon of February 8, field research teams were able to continue with GNSS observation. Because 3D positioning using GNSS was conducted based on the RTK GNSS technique, which enabled a high degree of accuracy within a short observation period, wind conditions did not have a significant effect on the final accuracy of the observations. On the other hand, most the LIDAR observations were conducted when there was no strong wind, no rain, and no snow to guarantee the stability of scanning operation. Moreover, since each scanning process lasted for an hour in windy conditions, a dual-axis compensator was operated with the terrestrial LIDAR to minimize the effect of winds. However, unpredictable strong wind occurred during scanning at station 4 and affected on recognizing co-registration targets.

**Table 2:** Activities during the research period.

Date	Activity	Date	Activity	Date	Activity
02/04	Installation of the base lines for GNSS observation	02/07	GNSS observation	02/10	Terrestrial LIDAR scanning
02/05	GNSS observation	02/08		02/11	
02/06	Time delay due to bad weather condition	02/09		02/12	Check for the observed data and instruments

**Figure 8:**
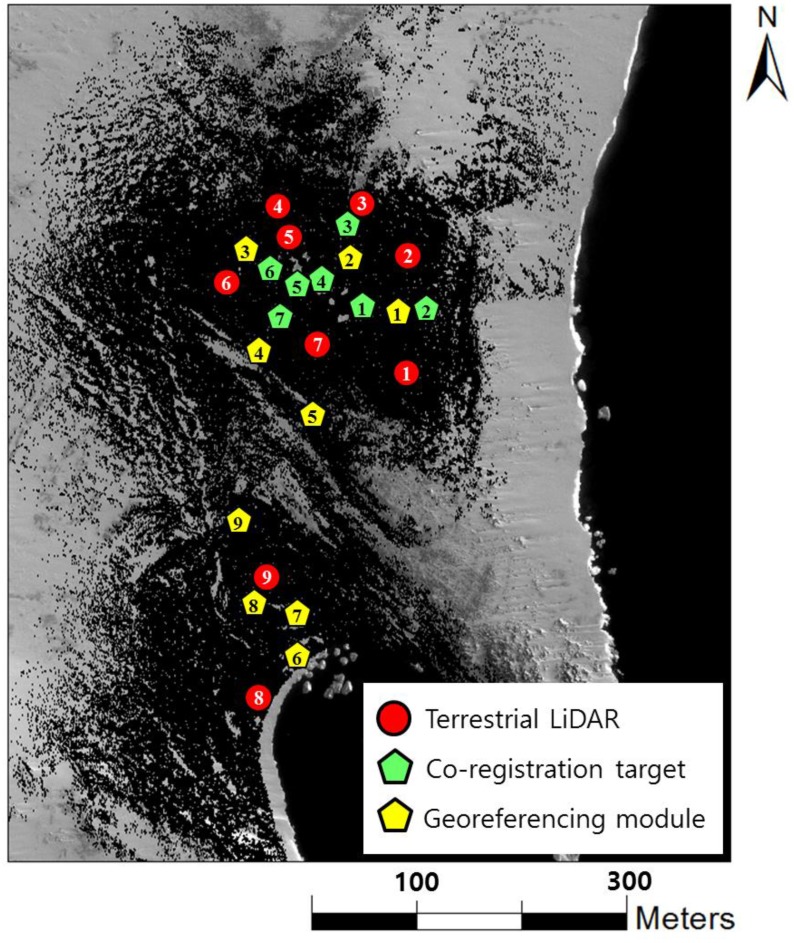
Location of stations for terrestrial LIDAR scanning, co-registration targets and georeferencing modules.

The selected locations of the terrestrial LIDAR stations, co-registration targets, and georeferencing modules for *in situ* observation are shown in Figure 8. Each pair of stations was able to observe at least three common targets and modules, and the distances between targets and stations were less than 70 m. During the nine sets of terrestrial LIDAR observations, installation of the instrument on a steep relief was avoided to ensure visibility while maximizing coverage and minimizing occlusion. However, an overlapped area could not be obtained between station 7 and 9 because of the steep relief of the research site. For this reason, co-registration and georeferencing of the point clouds at station 8 and 9 were performed separately.

**Figure 9:**
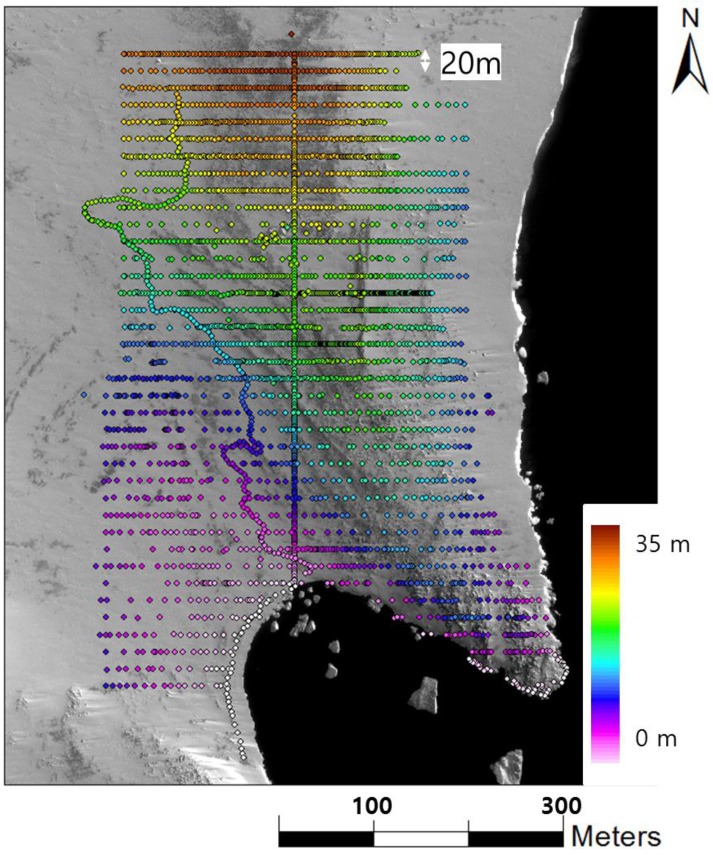
Location of check points observed by RTK GNSS.

Figure 9 shows the location of observed points using RTK GNSS. The 3D coordinates of 3134 check points were observed based on the RTK GNSS technique for the accuracy assessment of the point cloud. To minimize mistakes during RTK GNSS observation, 15 rows of points measurement spaced 40 m apart were conducted first, followed by the measurements of the remaining 15 rows. Measured 3D coordinates of check points were utilized for the accuracy assessment of the LIDAR point cloud and the DEM.

### 3.2. Co-Registration and Georeferencing

Co-registration and georeferencing results are summarized in Table 3. As shown in Table 3, at least three targets were recognized in the point cloud of each station except for station 4, and precise co-registration could be conducted using the targets except for the co-registration between stations 3 and 4.

**Figure 10:**
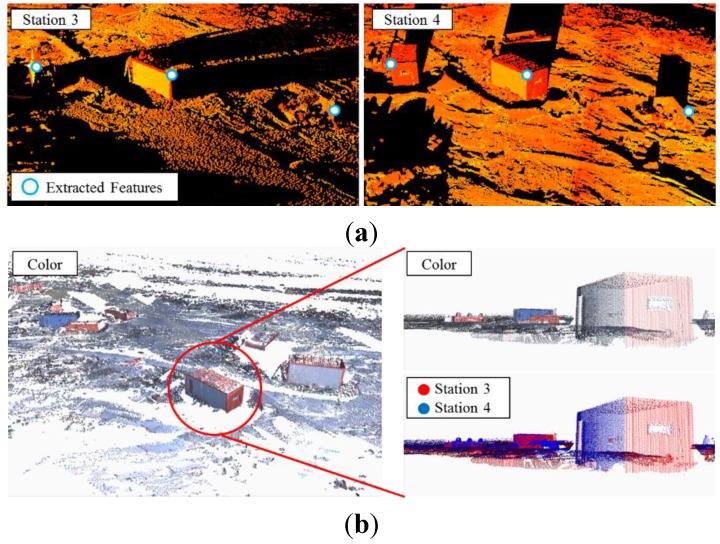
Co-registration between point clouds of stations 3 and 4: (**a**) examples of extracted natural feature points used for co-registration; (**b**) co-registration results (left and top-right: merged point cloud with color information, bottom-right: red colored point cloud and blue-colored point cloud were achieved at stations 3 and 4, respectively).

Since no planar target was detected in the point cloud observed in station 4 because of an unpredictable blizzard on February 10, natural feature points were used for initial co-registration between the point clouds of stations 3 and 4, and the ICP algorithm was then applied to improve the precision of co-registration. Figure 10 represents the extracted feature points in overlapped point clouds and the result of co-registration between a pair of point clouds observed at station 3 and station 4. Initial co-registration parameters were estimated using the extracted features. Then, the ICP algorithm was applied based on the estimated parameter. As shown in Figure 10b, although no artificial target was used, co-registration was successful. The average RMSE of co-registration could be achieved by 5 mm.

**Table 3:** Results of co-registration and georeferencing.

Station	Distance (m)	Overlapped Area (m^2^)	Target ID	Co-Registration Precision (mm)	Georeferencing Precision (mm)	
					Before Filtering	After Filtering
st1–st2	126.223	15,907	1 ^c^ *	3	-	-
			1 ^g^ **	2	317	52
			2 ^c^	3	-	-
st2–st3	73.465	6522	1 ^c^	10	-	-
			2 ^g^	4	1164	Outlier *****
			3 ^c^	8	-	-
st3–st4	71.062	10,990	3 ^c^,2 ^g^, 4 ^c^	X ****	-	-
			ICP ***	6	-	-
st5–st6	77.581	10,447	5 ^c^	5	-	-
			6 ^c^	3	-	-
			3 ^g^	5	357	52
st6–st7	115.115	23,264	5 ^c^	2	-	-
			7 ^c^	3	-	-
			4 ^g^	4	827	Outlier
			5 ^g^	X	-	-
st8–st9 ******	117.381	25,555	6 ^g^	14	62	62
			7 ^g^	10	32	32
			8 ^g^	5	29	29
			9 ^g^	X	-	-
RMSE				5	398	45

* Co-registration target; ** Georeferencing module; *** Because of the blizzard during scanning at station 4, the co-registration between point clouds of station 3 and 4 was conducted based on extracted natural targets and the ICP algorithm; **** The center point of a target was not recognized in a point cloud; ***** Filtered as an outlier with a large error; ****** Co-registration and georeferencing of the point clouds obtained at station 8 and 9 were performed separately from other point clouds.

The coordinate systems of the co-registered point clouds were transformed into WGS 84 based on the 3D coordinates of the installed georeferencing modules and the average RMSE of georeferencing was 398 mm. Because of the instability due to strong winds during scanning, which may have led to locational errors of the modules, the second and fourth geolocation modules, which had large errors, were considered to be outliers and therefore removed. After filtering, the average precision of georeferencing reached 45 mm.

**Figure 11:**
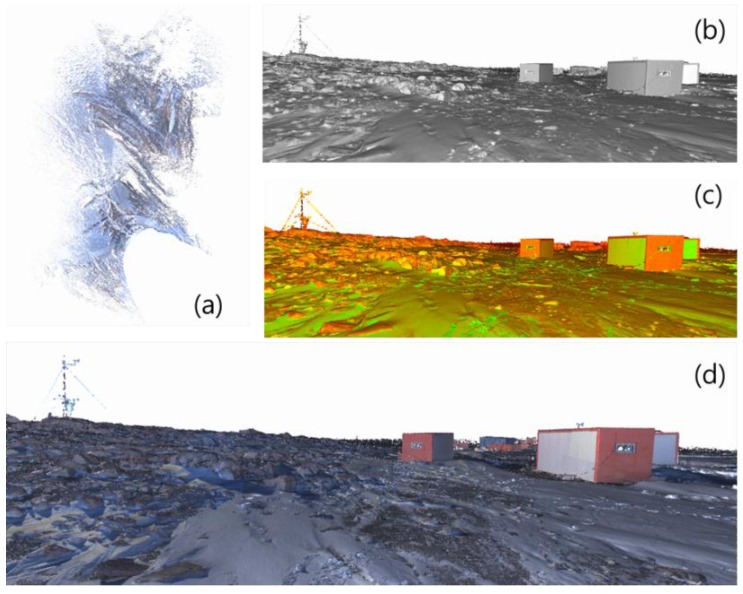
Visualization of point cloud data: (**a**) by plane view; (**b**) by gray-scale point cloud; (**c**) with intensity information of the received signal; (**d**) with color information obtained from the camera mounted on a terrestrial LIDAR system.

A total of 84,569,243 points were obtained during the nine rounds of terrestrial LIDAR scanning over the research site (Figure 11a). In addition, texture information was added to the point sets using a camera mounted on the terrestrial LIDAR system. Figure 11a shows an example of the point cloud with color information. This information (Figure 11d) helped in the identification of feature objects from the point clouds.

### 3.3. DEM Generation

**Figure 12:**
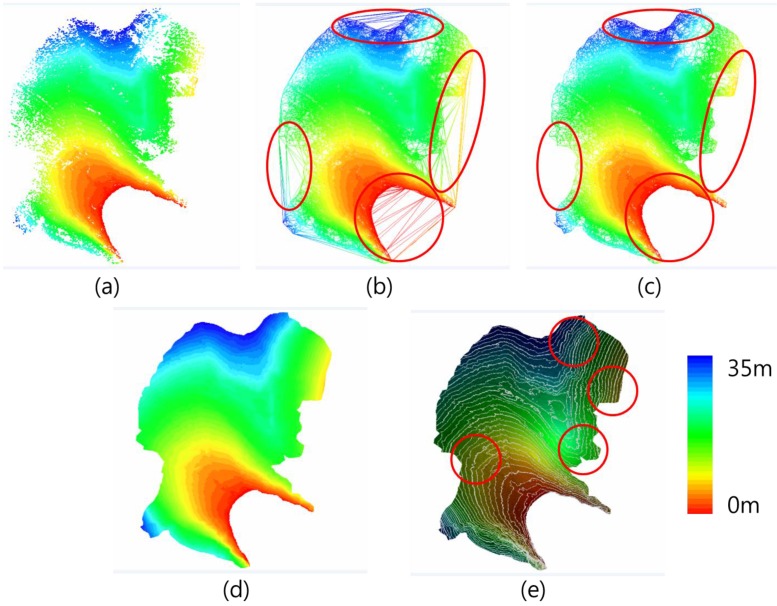
DEM generation from the merged point set: (**a**) merged point set; (**b**) TIN model (circle: Triangles with large areas or long edges); (**c**) filtered TIN model (circle: Erased unnecessary triangles in the original TIN model); (**d**) DEM of 50 cm spatial resolution; (**e**) contour lines of 1 m spacing overlapped on the TIN mesh (circle: Sparse point information, but a smooth contour was produced).

To produce an ancillary topographic map for the construction of Jang Bogo Station in Antarctica, we generated a high-resolution TIN, grid-based DEM, and contour lines from the georeferenced point cloud. Figure 12 shows the results of each step used to create the final DEM of the research site. After successful merging of point cloud data sets (Figure 12a) and removal of noisy points due to scanning, equipment, and research personnel on site, a TIN model (Figure 12b) was created. Further filtering was performed on the generated TIN model. As shown in Figure 12c, manual editing was required to remove unnecessary triangles with large areas and long edges on the boundary region of the research site. Using the filtered TIN model, a DEM with a 50 cm grid size (Figure 12d) and contour lines with 1 m line spacing (Figure 12e) were generated. As indicated in the circles of Figure 12e, notably, the final contour lines was smooth enough visually to represent the continuity and variability of the topography despite the sparse point information.

### 3.4. Accuracy Assessment

**Figure 13:**
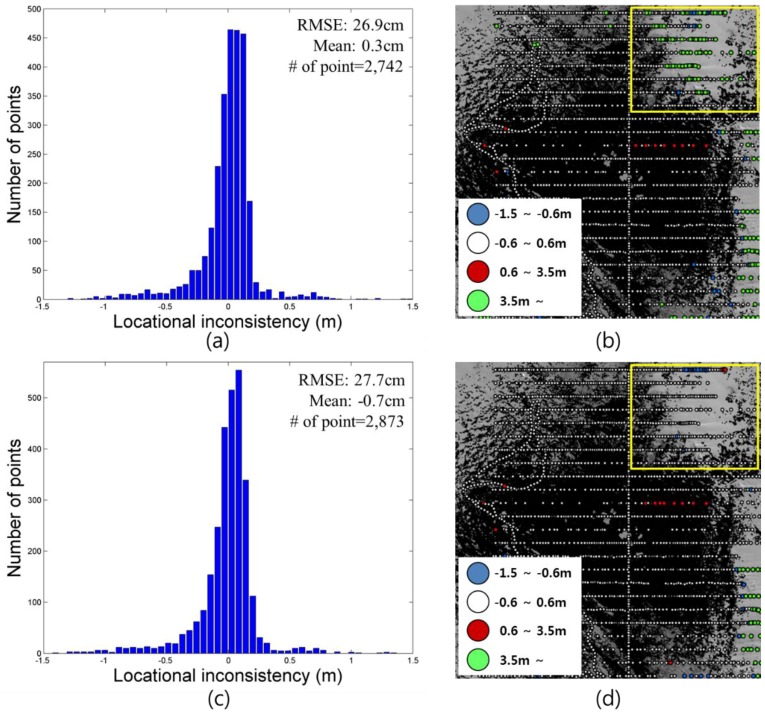
Elevation error: (**a**) and (**b**) between GNSS data and point cloud acquired using terrestrial LIDAR (yellow box: Occluded areas caused by high relief); (**c**) and (**d**) between GNSS data and generated DEM from scanning data (yellow box: Occluded area interpolated based on TIN).

Figure 13 shows a histogram and error map of the locational consistency between point clouds and checkpoints and between the generated 50 cm DEM and checkpoints. In a point cloud, the 3D coordinates of a checkpoint and the point closest to it were compared. If the distance between the checkpoint and nearest point was longer than 3.5 m, the checkpoint was excluded from the point group for calculating the histogram (Figure 13a,c) and represented by a green circle (Figure 13b,d). As shown in Figure 13a,c, the mean error and RMSE between the elevation values of the point cloud and checkpoints were 0.3 cm and ±26.9 cm, respectively. The mean error and RMSE between the elevation values of the DEM and GNSS observations were −0.7 cm and ±27.7 cm, respectively. Although the terrestrial LIDAR system can observe large areas within a short period of time with a high degree of accuracy, as shown by the green circles in the yellow box of Figure 13b, steep relief caused occluded areas and reduced accuracy. However, as shown in the boxes of Figure 13b,d, points in occluded areas could be interpolated in the DEM from the TIN-based linear interpolation and histograms in Figure 13a,c, which represent similar trends in the elevation errors.

**Figure 14:**
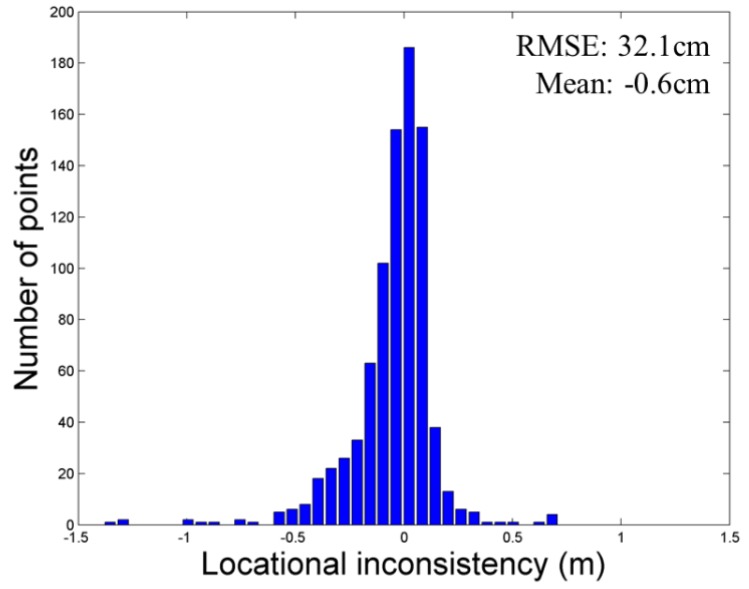
Elevation error of the point cloud acquired at station 4.

Figure 14 shows the elevation error for the point cloud acquired at station 4. Locational inconsistency between the GNSS data and the point cloud of station 4 was only ±32.1 cm of RMSE, and the error trend was similar to that of the whole point cloud dataset. If there are adequate feature points for co-registration and a sufficiently overlapped area between a pair of point clouds, the co-registration method based on feature points extracted in overlapped point clouds and the ICP algorithm can achieve accuracy as high as that of co-registration using artificial targets.

**Figure 15:**
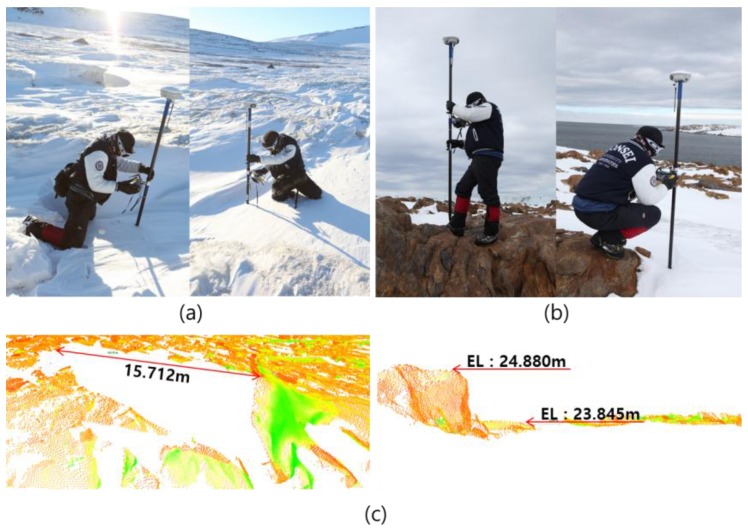
Causes of location inconsistency between GNSS observation and scanning data: (**a**) snow-covered land surface; (**b**) small rocks; **(c**) occluded areas in point clouds.

Considering the environment of Terra Nova Bay, we can deduce that the elevation errors were caused by the complexity of the scanning site and the snow covering the land surface. Since terrestrial LIDAR scanned snow-covered land surfaces, the elevation of the scanning data was higher than the results of the GNSS observation (Figure 15a). In addition, small rocks and steep relief resulted in occlusion areas in point cloud (Figure 15b,c).

## 4. Conclusions

In the operation of a terrestrial LIDAR system in Antarctica, harsh weather conditions can affect stability and decrease the accuracy of observed data. Since the low temperatures in Antarctica can cause device malfunction, preliminary tests to verify stability of the LIDAR system and practical solutions to ensure LIDAR functionality are required. Furthermore, irregular strong winds and complex terrain covered with thick snow make co-registration and georeferencing of point clouds difficult. Therefore, establishing methods of reducing the time requirement for LIDAR operation and ensuring quality of the final results are necessary.

This study proposes and demonstrates the following practical solutions for observations using a terrestrial LIDAR system in Antarctica: (1) a lagging cover with a heating pack to maintain the temperature of the terrestrial LIDAR system; (2) co-registration using square planar targets and two-step point-merging methods based on extracted feature points and the ICP algorithm; and (3) the georeferencing module consisting of an artificial target and GNSS. From the *in situ* observations, it is evident that the proposed methods and devices ensured stable operation of the terrestrial LIDAR system and accurate observations. Despite the complex terrain, irregular winds, and thick snow cover, there was only ±27.7 cm of RMSE between the checkpoints observed by RTK GNSS and the DEM obtained from the LIDAR scanning data.

The terrestrial LIDAR system has several advantages over GNSS observation. Terrestrial LIDAR is able to cover a large area in a single observation with high precision and point density and exhibited much higher productivity than did RTK GNSS. The inclusion of color information using the camera mounted on the LIDAR system can also improve the interpretation of objects in a point cloud. Furthermore, a TIN model, a DEM with 50 cm resolution, and 1 m contour lines were produced using the terrestrial LIDAR scanning data. Based on the high-resolution DEM and contour lines produced in this study, the Jang Bogo Station in Antarctica was successfully constructed in February 2014.
